# Alternative splicing of flowering time gene *FT* is associated with halving of time to flowering in coconut

**DOI:** 10.1038/s41598-020-68431-2

**Published:** 2020-07-15

**Authors:** Wei Xia, Rui Liu, Jun Zhang, Annaliese S. Mason, Zhiying Li, Shufang Gong, Yazhu Zhong, Yajing Dou, Xiwei Sun, Haikuo Fan, Yong Xiao

**Affiliations:** 1 National Engineering Research Center of Coconut/Coconut Research Institute, Chinese Academy of Tropical Agricultural Sciences, Wenchang, People’s Republic of China; 2 College of Tropical Crops, Hainan University, Haikou, People’s Republic of China; 3 Department of Plant Breeding, IFZ Research Centre for Biosystems, Land Use and Nutrition, Justus Liebig University Giessen, Heinrich-Buff-Ring 26-32, 35392 Giessen, Germany; 4 Hainan Key Laboratory for Biosafety Monitoring and Molecular Breeding in Off-Season Reproduction Regions, Haikou, People’s Republic of China

**Keywords:** Plant breeding, Plant signalling

## Abstract

Coconut palm has two distinct types—“tall” and “dwarf”—which differ morphologically. Tall coconut varieties need 8–10 years to start flowering, while dwarf coconut varieties only require 3–5 years. We compared seedling and reproductive stage transcriptomes for both coconut types to determine potential molecular mechanisms underlying control of flowering time in coconut. Several key genes in the photoperiod pathway were differentially expressed between seedling and reproductive leaf samples in both tall and dwarf coconut. These genes included *suppressor of overexpression of constans* (*SOC1*), *flowering locus T* (*FT*), and *Apetala 1* (*AP1*). Alternative splicing analysis of genes in the photoperiod pathway further revealed that the *FT* gene produces different transcripts in tall compared to dwarf coconut. The shorter alternative splice variant of *FT* [which included a 6 bp deletion, alternative 3′ splicing sites (A3SS)] was found to be exclusively present in dwarf coconut varieties but absent in most tall coconut varieties. Our results provide a valuable information resource as well as suggesting a probable mechanism for differentiation of flowering time onset in coconut, providing a target for future breeding work in accelerating time to flowering in this crop species.

## Introduction

Coconut palm (*Cocos nucifera* L., 2n = 32) belongs to the monocotyledonous family Arecaceae (Palmaceae) and is an important tropical oil and fruit crop. Presently, coconut palm is cultivated in 93 countries, including Central and South America, East and West Africa, Southeast Asia and the Pacific island, where it accounts for over 12 million hectares^1^. Coconut palm is divided into “tall” and “dwarf” types based on distinct morphological and breeding features between the two groups. Tall coconuts take 8–10 years to complete the vegetative to reproductive transition, can grow to a height of about 20–30 m, and have medium-to-large nuts. Tall coconuts are hardy and can adapt to a wide range of environmental conditions. Dwarf coconuts are early-flowering (4–6 years after planting), can grow to a height of about 10–15 m, and can produce a number of small nuts^2^. The large difference in flowering time between these two types is of particular interest for breeding and production: flowering time limits not only generation speed but also time to first harvest after planting, such that shorter flowering times are almost always desirable. However, to date little is known about regulation of flowering time in coconut.

In the last several decades, a great deal of work has been done in elucidating the molecular mechanisms underlying the transformation from vegetative growth to reproductive growth in plants. Four flowering regulation pathways have been described in model plant *Arabidopsis thaliana*: the photoperiod pathway^3^, the vernalization pathway^4^, the gibberellin pathway^5^ and the autonomous pathway^6^. Time to flowering in *Arabidopsis thaliana* is predominantly determined by expression of the *Flowering Locus T* (*FT*) gene^7^. In *Arabidopsis thaliana FT* is negatively regulated by expression of *Flowering Locus C* (*FLC*), which is a transcriptional repressor, and positively regulated by expression of *Constans* (*CO*), which is a transcriptional activator^7^. Overexpression of *CO* up-regulates *FT* and causes an early flowering phenotype^8^, as does direct overexpression of *FT*^9,10^ *CO* is itself negatively regulated by *Suppressor of Overexpression of Constans 1* (*SOC1*) and *PhyB*^11^, the latter of which is a photoreceptor^12^. *PhyB* loss of function mutants also therefore result in early flowering^12^. Flowering time phenotypes have also been observed as a result of mutation of other photoreceptor genes, such as *CRY2*^13^. In total at least 29 major genes are involved in regulation of flowering time in *Arabidopsis thaliana* , including *Flowering Locus D* (*FLD*) and *Leafy* (*LFY*)^14–18^.

The function of *FT* has also been well studied in many species other than model plant *Arabidopsis thaliana*. In rice, a total of thirteen *FT*-like homologs were identified, of which three FT-like genes can promote flowering (*OsFTL1*-*OsFTL3*)^18–21^. Of these genes, *OsFTL2* was found to be a mobile signal that moves from the leaf to the shoot apical meristem to induce flowering^22,23^. In barley, a total of five *FT*-like genes were found, of which *HvFT1* was highly expressed under long-day conditions in transition from vegetative to reproductive growth, *HvFT3* was identified as a candidate gene under a major flowering-time QTL, *HvFT2* and *HvFT4* were highly expressed at later developmental stages and *HvFT5* showed low expression level under short-day conditions^24^; highlighting the possible complexity of regulatory mechanisms involving multiple *FT* copies. In rapeseed, which has a history of polyploidy, 164 gene copies were identified from 29 core *Arabidopsis* flowering time gene homologs, an average of 4.7 copies per *Arabidopsis* gene^25^; 101 genome regions in this species were significantly associated with flowering time^26^ and 12 haplotypes differentiated winter and spring-flowering rapeseed^7,27^. By contrast, although a total of four *FT*-like genes were isolated in poplar, only one (*PtFT2*) was shown to shorten juvenile phase and promote seasonal flowering^28^.

In coconut palm, we have as yet no information about the molecular basis of flowering time control, despite the importance of the flowering time trait in this long-lived agricultural tree species. In the present study, we used comparative transcriptomics to elucidate the molecular mechanisms underlying the differences in flowering time observed between tall and dwarf coconut types. Coconut orthologs of 174 *Arabidopsis* genes belonging to the flowering-time pathway were identified and characterized for expression in different tissues from seedling and reproductive stages. Among these genes, we detected and validated alternative splicing of *CnFT* transcripts as the putative causal mechanism for the observed flowering time difference between tall and dwarf coconut types.

## Materials and methods

### Plant materials and RNA isolation

The leaf tissues of seedling and mature stage of twococonut plants were used for RNA-seq: 10-year-old Yellow Dwarf (YD) individuals; 1-year-old YD seedlings; 10-year-old Hainan Tall (HT) individuals and 1-year-old HT seedlings. Each sample included a mixture of two biological replicates. The eight coconut individuals were growing in the coconut germplasm resource of the Coconut Research Institute of the Chinese Academy of Tropical Agricultural Sciences. The average temperature and humidity in this location are approximately 23.9 °C and 89%. Total mRNA was extracted from spear leaves of these coconut individuals according to the MRIP method^29^.

### Illumina sequencing

Purified mRNA was separately fragmented with divalent cations under increased temperature. These short fragments were taken as templates to synthesize the first-strand cDNA using hexamer primers and superscript III (Invitrogen, Carlsbad, CA, USA). Second-strand cDNA was then synthesized in a solution containing buffer, dNTP, RNaseH and DNA polymerase I and subsequently purified using a QiaQuick PCR extraction kit (Qiagen). EB buffer was used to resolve these short fragments for end reparation and poly (A) addition. The sequence adaptors were linked to two ends of short cDNA sequences and suitably sized cDNA fragments were selected out for PCR amplification based on the agrose gel electrophoresis results. Finally, the library established was sequenced using an Illumina Hiseq 200. The paired-end library was developed according to the paired-end sample Preparation kit protocol (Illumina, USA).

### Real-time qPCR assays

Real-time qPCR was performed following a standard SYBR Premix Ex Taq Kit (TaKaRa) protocol in 96-well optical plates (Axygen) using a volume of 10 μl. The primer pairs were designed by using Snapgene Viewer 1.0 software (Table 1). The reactions were incubated in 0.2 ml tubes of a Mastercycler Ep Realplex4 (Eppendorf) machine as follows: 95 °C for 5 s, 55 °C for 15 s and 68 °C for 20 s. The procedure ended by a melt-curve ramping from 60 to 95 °C for 20 min to check the PCR specificity. All qPCR reactions were carried out in biological and technical triplicates. The final Ct values were the means of nine values. The comparative expression levels of the selected expressed sequences were normalized to that of *actin*.

**Table 1:** Primer sequence for real-time qPCR.

Gene ID	Forward primer	Reverse primer
AP1_CCG008583.1	AGGGAAGACGGAGGTGAGAC	GGGAGAGAAGACGATGAGGG
AP1_CCG008584.1	GGGGAAGACGGAGGTGAGACG	CCCTTGGGGGAGAAGACGATG
AP1_CCG022175.1	CCAGGTCTCCGTCGTCATCT	CTCTCGTGCTTGGCATCCC
AP1_CCG027514.1	AGGGGGAAGACGGAGATGAA	GAGAAGACGATGACGGCGAC
CO_CCG005523.2	CGAAGGGAGGGTTATTGGGG	GTCGCAGGTGACGCAGAGC
FT_CCG009539.1	TTCATCAGGTCCGCATTTCTT	AGGGTCCACCATCACGAGC
FT_CCG011565.1	GTGGTAGGGAGGGTCATAGGC	GGACAGTTGGAGGGTTGATAGC

### Calculating gene expression level

RPKM (Reads Per kb per Million reads) was used to calculate gene expression level using the following formula^30^:

$$RPKM= \frac{{10}^{6}C}{NL/{10}^{3}}$$

where C is the number of reads that exclusively aligned to one expressed sequence, N is the total number of reads that aligned to all expressed sequences, and L is the basic number in the CDS of the corresponding expressed sequence. The CDS and genome sequence (the first version of coconut genome) is available in the GigaDB database (GigaDB, RRID:SCR 004002, https://gigadb.org/dataset/100347).

The statistical significance of the differential expression was determined according to the method documented by Audic and Claveries^31^. The statistical significance *p(x)* can fit the Poisson distribution

$$p\left(x\right)= \frac{{e}^{-\lambda }{\lambda }^{x}}{x!}$$

Arabic alphabet x in the formula represents number of reads mapped to each unigene. λ refers to the number of real transcripts of the unigene.

When thousands of hypothesis tests are performed, the p-value suitable for a single test is not sufficient to guarantee a low rate of false discovery. Thus, an false discovery rate (FDR) control method was applied using multiple hypothesis testing to correct the *p*-value results^32^. Subsequently, the RPKM ratio was used to compute the fold change of gene expression for each pair of samples simultaneously. The differentially expressed genes were selected using a threshold of FDR ≤ 0.001 and an absolute value of log_2_^ratio ^≥ 1^33^.

#### The functional annotation of differential expressed genes

The predicted genes were used for BLAST and annotation against NCBI non-redundant sequence database (Nr) at a cut-off value of 10^–5^, The differential expressed genes were also aligned by BLASTX to protein databases such as GO, KEGG, Swissprot, and InterPro database (transcription factor), and PRG database (PRG). The Blast2GO program was used to obtain GO annotations for differential expressed genes. The WEGO software was then used to perform GO functional classification of differential expressional genes. Meanwhile, Blastall software was used to align differential expressed genes against KEGG (Kyoto Encyclopedia of Genes and Genomes) database. KEGG enrichment was performed by using phyper of R package. Enrichment of KEGG pathway were performed at a cut-off FDR of 0.01.

### Detection of alternative splicing

**Figure 1:**
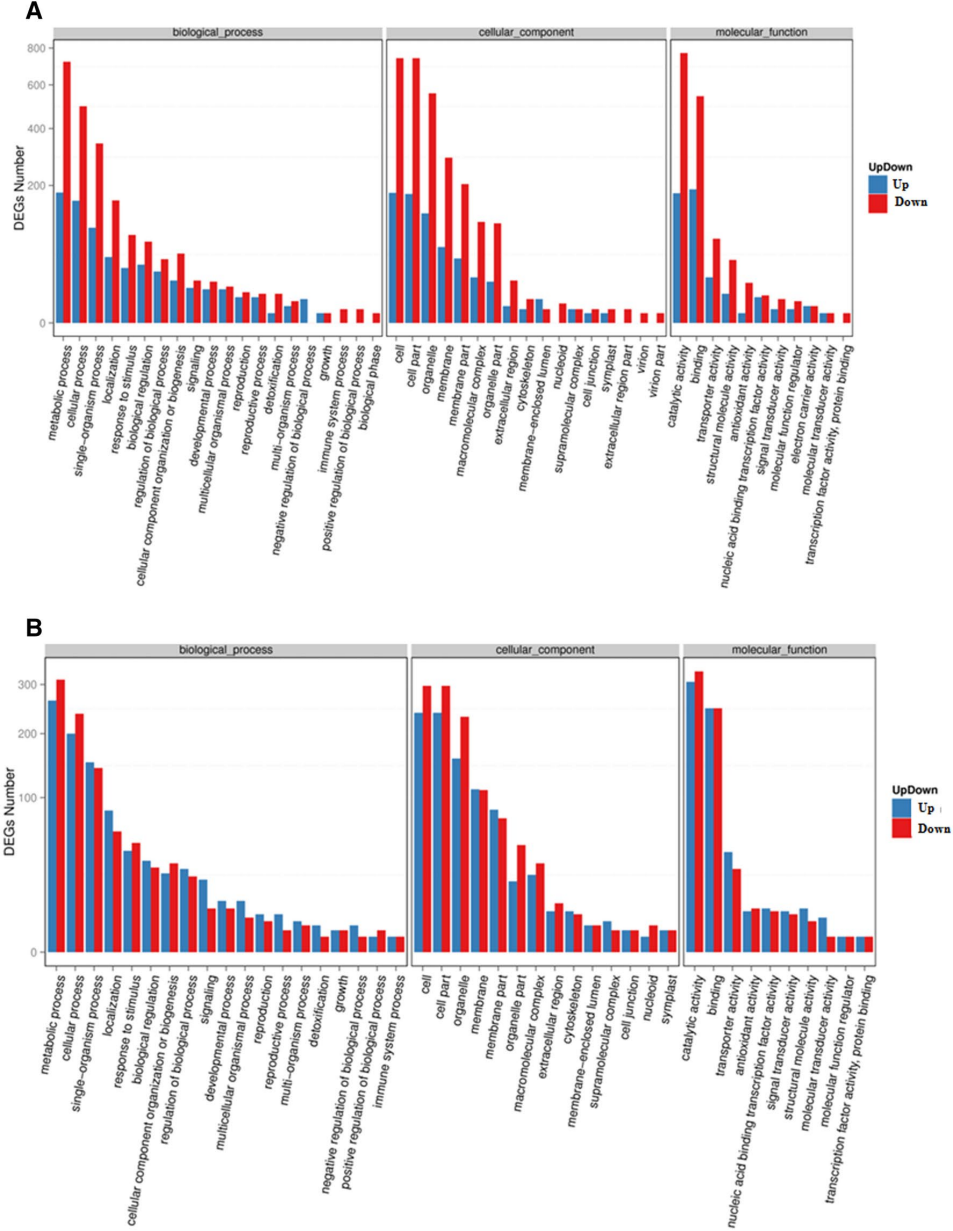
Gene ontology annotation of differentially expressed genes between seedling and mature trees of (**A**) tall coconut and (**B**) dwarf coconut.

We used rMATS 4.0.1 software to detect alternative splicing events based on RNA-seq data and coconut genome sequence (GigaDB, RRID:SCR 004002, https://gigadb.org/dataset/100347)^34^. The p-value and False Discovery Rate (FDR) were used to determine the isoform ratio of one gene at a cut-off PDR of 0.05. Detailed information is shown in Fig. 1. To exclude the influence of insertion/deletion variation in AS detection, we used STAR to map RNA-seq clean reads and GATK4 to call indel variations. The detected AS events that overlapped with transcript indel variations were filtered out for further analysis.

### PCR amplification and sequencing of 20 coconut individuals

Primers were designed according to the exon sequences flanking alternative splicing junctions using Snapgene Viewer 1.0 software. The primer sequences were listed in Table 2. PCR amplifications were performed in 50 μl reaction mixures containing a 500 ng cDNA sample from the oil palm mesocarp, 1 × PCR buffer, 2 mM MgCl_2_, 5 U of TaqDNA polymerase (TaKaRa, China), 0.5 μM of each primer, and 0.2 mM dNTP mix. The PCR program included denaturation at 98 °C for 30 s; followed by 30 cycles at 98 °C for 10 s, 55 °C for 30 s, and 72 °C for 60 s; and a final extension at 72 °C for 5 min. The PCR products were electrophoretically visualized on a 1% agarose gel.

**Table 2:** Primer sequences of FT gene for alternative splice site.

Primer name	Primer sequence
FT_AS_475-F	GTGGTAGGGAGGGTCATAGGC
FT_AS_475-R	GGGCATTGAAGTAGACCGCAG

## Results

### Transcriptome sequencing for tall and dwarf coconut

Approximately 6.6 Gb of unmapped clean reads were generated for each of four samples: tall coconut seedlings, tall coconut mature trees, dwarf coconut seedlings and dwarf coconut mature trees (Table 3). All clean reads have been deposited in the European Nucleotide Archive (Submission Number: PRJEB34852). After mapping all clean reads to the coconut reference genome using the software HISAT, a total of 36,558 transcripts were predicted. Among them, a total of 7,037 transcripts were predicted to be non-coding RNA. The remaining 29,521 were identified as protein-coding transcripts, 25,114 (85%) of which were consistent with previously known proteins predicted based on the coconut genome and the remaining 4,407 of which were novel protein-coding transcripts.

**Table 3:** Transcriptome raw data obtained from seedlings and mature tall and dwarf coconut trees.

	Clean reads (Gb)	Number of reads (million)	Q20 (%)
**Tall coconut**			
Seedling	6.59	65.94	97.58
Mature	6.57	65.74	97.52
**Dwarf coconut**			
Seedling	6.63	66.34	97.62
Mature	6.68	66.75	97.5

### Analysis and functional annotation of differential expression genes between tall and dwarf coconut

Global expression changes were identified between seedlings and mature trees of tall and dwarf coconut using the software program PossionDis. Compared to seedling period of tall coconut, 1729 transcripts were up-regulated and 4,150 were down-regulated (*abs* (log_2_^Foldchange^) < 1 or *FDR* > 0.001) in mature period of tall coconut. Meanwhile, compare to seedling period of dwarf coconut, 1964 transcripts were up-regulated and 2009 were down-regulated (*abs* (log_2_^Foldchange^) < 1 or *FDR* > 0.001) in seedling period of dwarf coconut.

**Figure 2:**
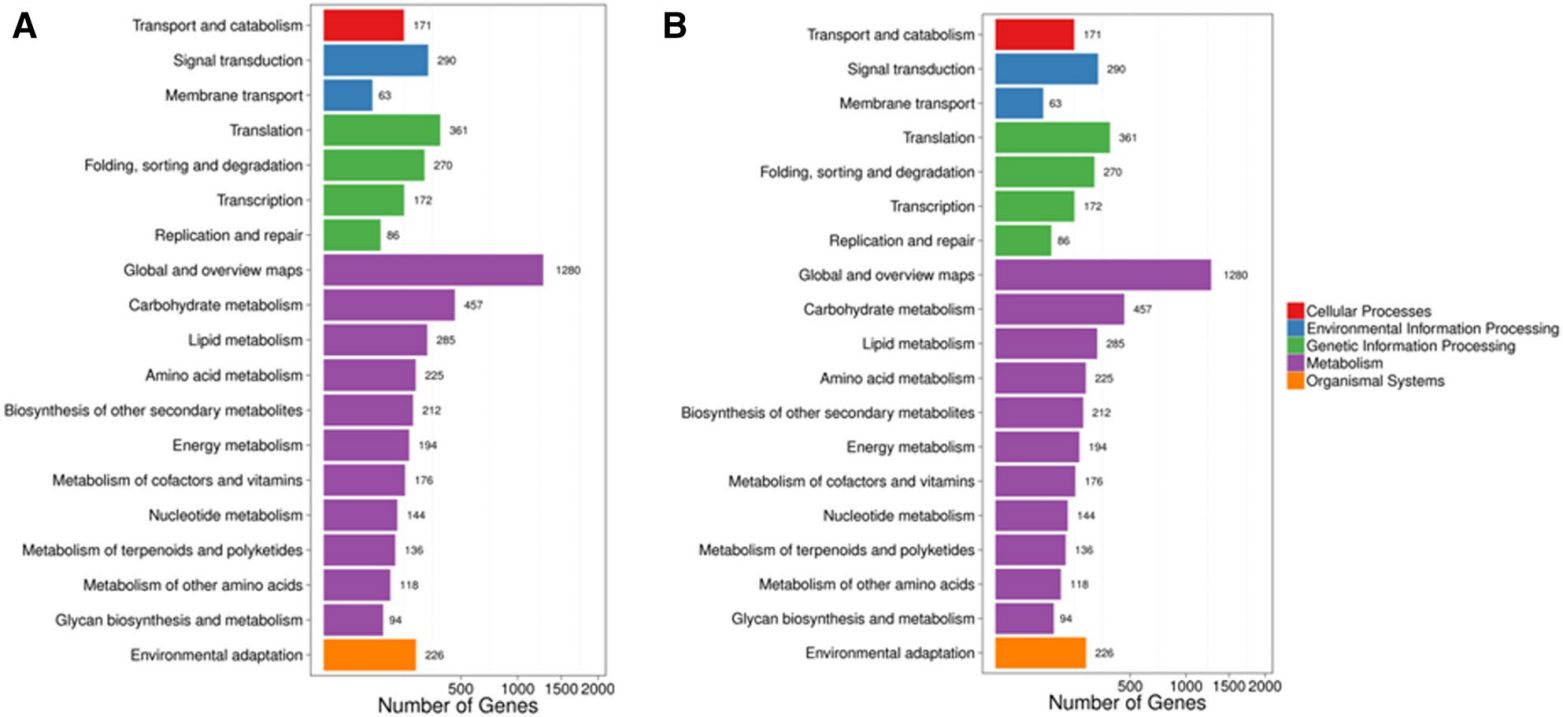
KEGG annotation of differentially expressed genes between seedling and mature trees of (**A**) tall coconut and (**B**) dwarf coconut.

In order to annotate differentially expressed unigenes, unigene sequences were aligned against the NCBI non-redundant (Nr) protein database using a cut-off E-value of 10^–5^ (Fig. 2). A large proportion of differentially expressed unigenes were assigned into the metabolic process (tall coconut: 902 unigenes and dwarf coconut: 576 unigenes) and the cellular process (tall coconut: 655 unigenes and dwarf coconut: 438 unigenes) categories for the “biological process” categories, cell (tall coconut: 921 unigenes and dwarf coconut: 537 unigenes) and cell part (tall coconut: 918 unigenes and dwarf coconut: 537 unigenes) in the “cellular component” categories, and catalytic activity (tall coconut: 949 unigenes and dwarf coconut: 636 unigenes) and binding (tall coconut: 733 unigenes and dwarf coconut: 498 unigenes) in the molecular function categories. Only down-regulated unigenes were detected in some functional categories in mature trees of dwarf coconut compared to dwarf coconut seedlings, including immune system processes, positive regulation of biological processes and biological phases under the biological processes categories, membrane-enclosed lumen, extracellular regions, and virion in the cellular component categories, and transcription factor activity and protein binding in the molecular function categories. By contrast, in each of these functional categories, both up-regulated and down-regulated unigenes were detected in mature trees of tall coconut compared to tall coconut seedlings.

**Figure 3:**
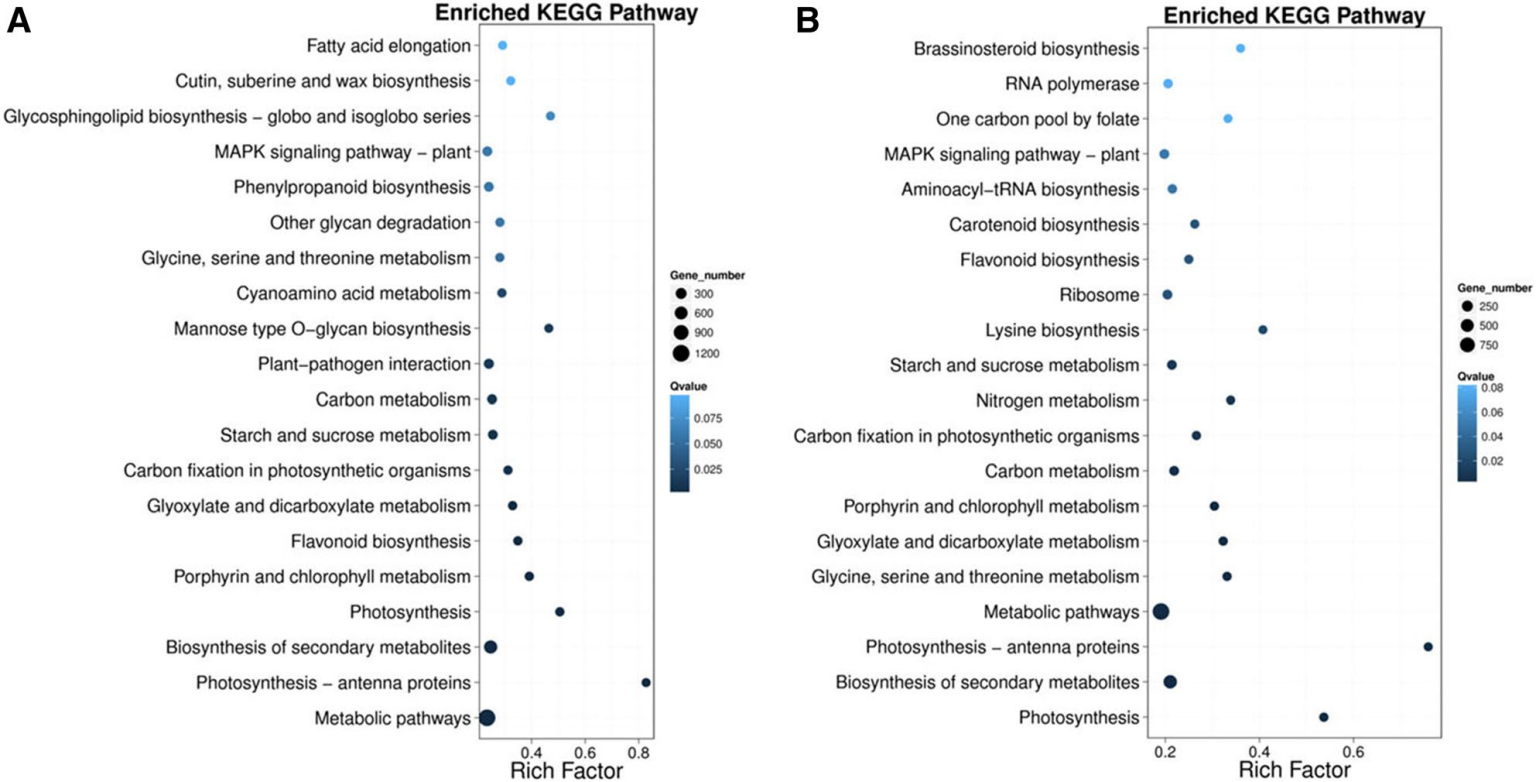
Enriched KEGG (Kyoto Encyclopedia of Genes and Genomes) pathway of differentially expressed genes between seedlings and mature trees in (**A**) tall coconut and (**B**) dwarf coconut.

In order to further identify the active biochemical pathways of differentially expressed unigenes, we mapped these differential expressed genes (*abs* (log_2_^Foldchange^) < 1 or *FDR* > 0.001) to the reference canonical pathways in the Kyoto encyclopedia of genes and genomes (KEGG). A total of 2,580 differential expressed genes between seedling and mature stage of tall coconut were mapping the 115 KEGG pathways (Fig. 2). Moreover, in dwarf coconut, 1,704 differential expressed genes were mapped onto 113 KEGG pathways. Enrichment results for KEGG pathways showed that the highest enrichment factors were detected in the photosynthesis-antenna protein pathway in dwarf coconut, followed by the photosynthesis pathway (Fig. 3). Meanwhile, in tall coconut, the highest enrichment factors were detected in the photosynthesis-antenna protein pathway, followed by Mannose type O-glycan biosynthesis.

Meanwhile, Transcription factors were also predicted by aligning differential expressed genes with COG database. A total of 685 differential expressed genes in tall coconut were identified and mapped onto 46 gene families. While, in dwarf coconut, 509 differential expressed genes were identified to be transcription factors and mapped into 45 gene families. Meanwhile, differential expressed genes were also blasted against PRG database. A total of 340 differential expressed genes were identified to be resistant genes in tall coconut. Moreover, in dwarf coconut, 208 differential genes were identified to resistant genes.

### Identification of candidate genes involved in flowering-time pathways and their expression profiles

In order to identify candidate genes involving in flowering-time pathways, protein sequences of 174 *Arabidopsis* genes involving in flowering time were downloaded from the National Center for Biotechnology information (NCBI). Detailed information for these genes is listed in Supplementary Table 1. Subsequently, these *Arabidopsis* protein sequences were aligned using BLAST with the “tblastn” function against the CDS (coding sequences) database of coconut^35^. A total of 198 genes in coconut were identified as homologues of flowering-time genes in *Arabidopsis*. Detailed information for these coconut genes is listed in Supplementary Table 2. The 198 genes were classified into the four flowering-time pathways [photoperiod pathway, autonomous pathway, vernalization pathway, and gibberellin (GA) pathway] based on their *Arabidopsis* annotations.

**Figure 4:**
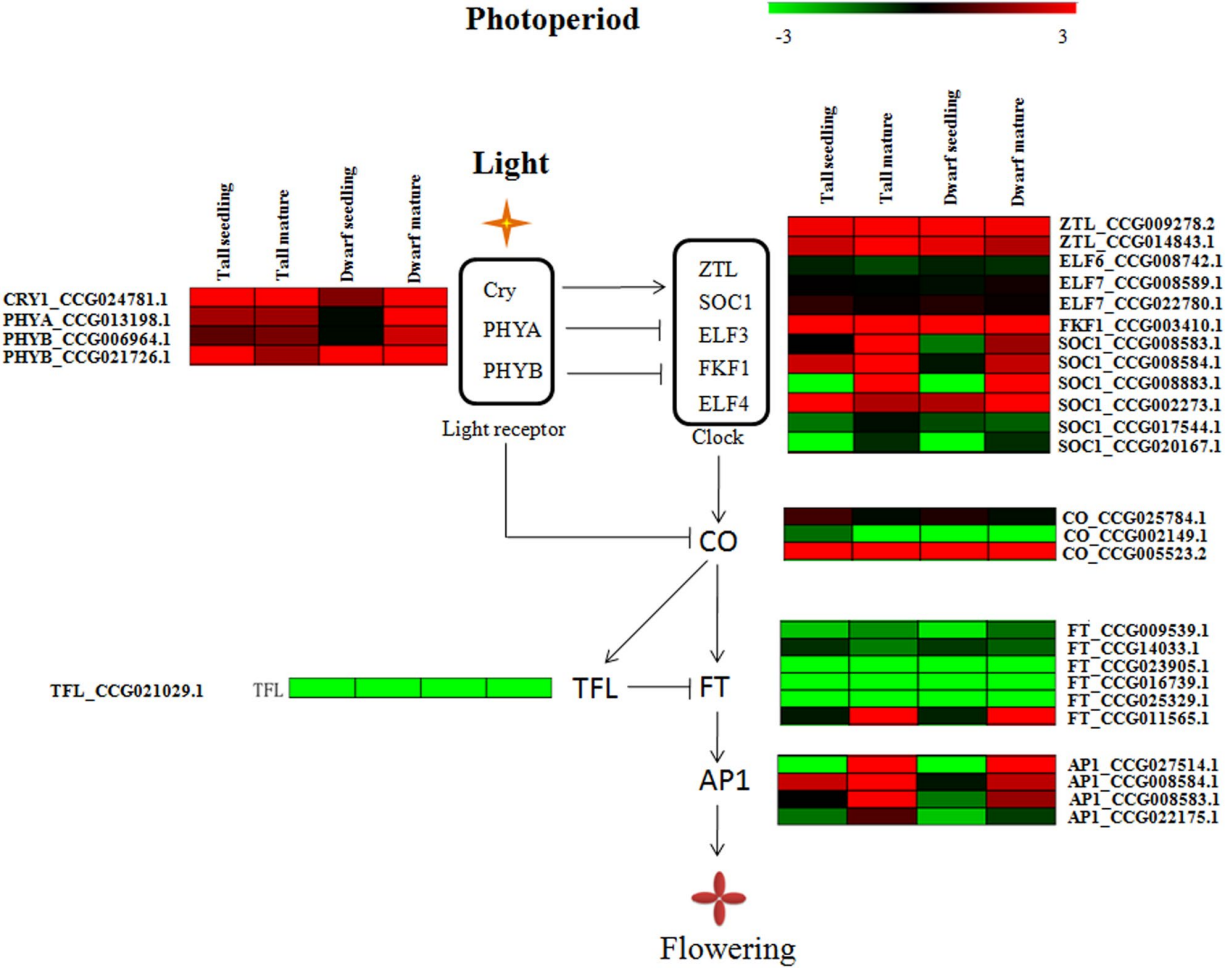
Gene expression changes involving the photosynthesis pathway which govern flowering time between seedlings and mature trees in tall and dwarf coconut.

From this data we identified four light receptor genes, including one copy of *CRY1* (*cryptochrome 1*), one copy of *PHYA* (*phytochrome A*), and two copies of *PHYB* (*phytochrome B*), all of which showed high expression levels both in seedlings and mature trees of tall and dwarf coconut (Fig. 4) and are known to regulate clock genes. We identified 12 putative clock genes in the coconut genome: two *ZTL* (*zeitlupe*), one *ELF6* (*early flowering 6*), two ELF7 (*early flowering 7*), one *FKF1* (*flavin binding F-box 1*), and six *SOC1* genes. Among them, three *SOC1* genes (CCG008583, CCG008584, and CCG008883) showed higher expression levels in mature trees compared to seedlings in both tall and dwarf coconut. CCG008883 in particular had FPKM values of 0.22 and 0.34 in tall and dwarf coconut seedlings respectively, but FPKM values of 55.16 and 50.12 in mature trees of tall and dwarf coconut respectively. *SOC1* is known to induce *CO* gene expression in *Arabidopsis*: we also found three *CO* orthologs in coconut (CCG025784, CGG002149, and CCG005523), one of which (CCG005523) was highly expressed at both seedling and reproductive stages of tall and dwarf coconut, while the other *CO* genes had low expression levels at both developmental stages. *CO* genes are known up-regulators of the *Flowering Locus T* (*FT*) gene, which triggers the transition from vegetative to reproductive growth. We identified six *FT* homologs in coconut: CCG009539.1, CCG014033.1, CCG023905.1, CCG016739.1, CCG025329.1, and CCG011565.1. Among them, CCG011565.1 was more highly expressed at the reproductive stage than the seedling stage in both tall and dwarf coconut. Finally, *FT* can also enhance the expression of the *AP1* gene. We found four *AP1* homologs in coconut: CCG027514.1, CCG008584.1, CCG008583.1, and CCG022175.1. Among them, higher expression was detected for CCG027514.1 in mature trees compared to in seedlings of tall and dwarf coconut.

**Figure 5:**
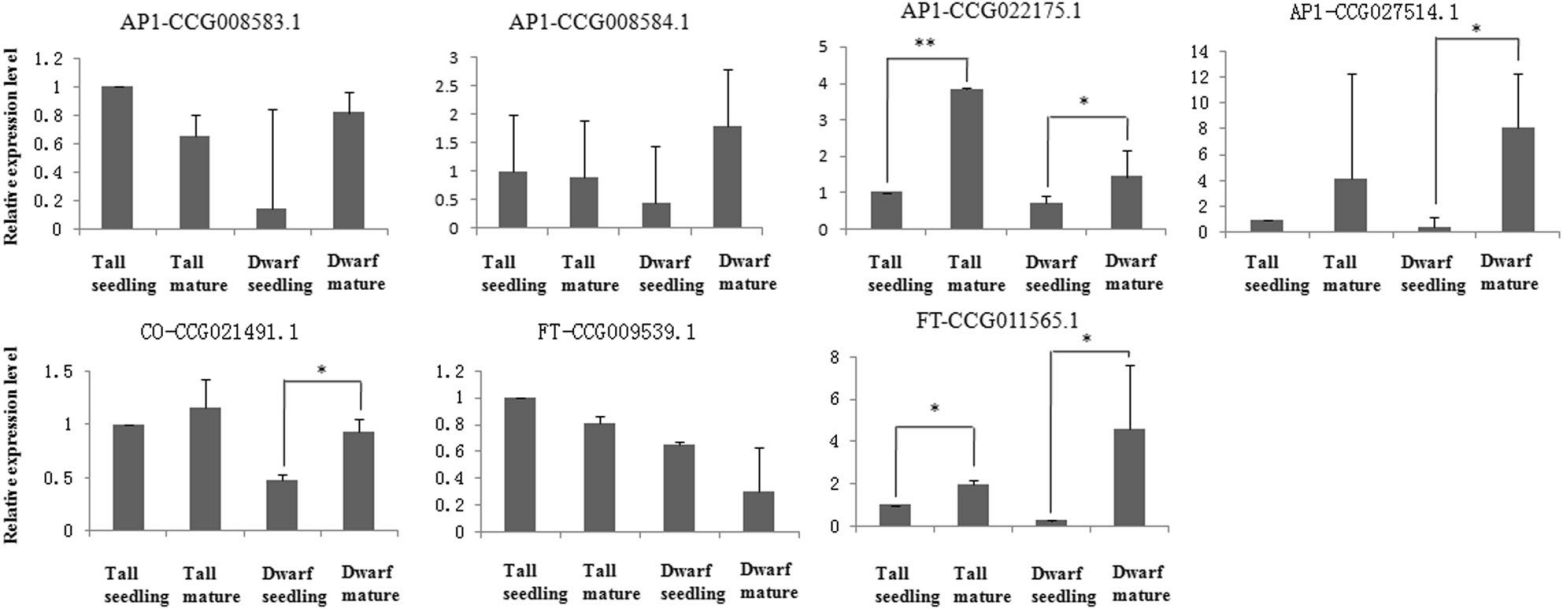
Quantitative real-time PCR validation of candidate genes involved in the photosynthesis pathway, including *AP1*, *FT* and *CO.* *Significant difference of gene expression between mature and seedling period of tall and dwarf coconut (*P* < 0.05). **High significant difference of gene expression between mature and seedling period of tall and dwarf coconut (*P* < 0.001).

Meanwhile, quantitative real-time PCR (qPCR) was used to validate the gene expression profile of several genes involved in the photoperiod pathway: *CO* (CCG021491), *FT* (CCG009539 and CCG011565), and *AP1* (CCG008583, CCG008584, CCG022175, and CCG027514) gene copies (Fig. 5). The qPCR results were similar to the transcriptome data results. Higher expression levels in *CO* (CCG021491), *FT* (CCG011565), and *AP1* (CCG022175 and CCG027514) were detected in mature trees compared to in seedlings of tall and dwarf coconut.

### Alternative splicing of Flowering Locus T (FT) and association with flowering time

**Figure 6:**
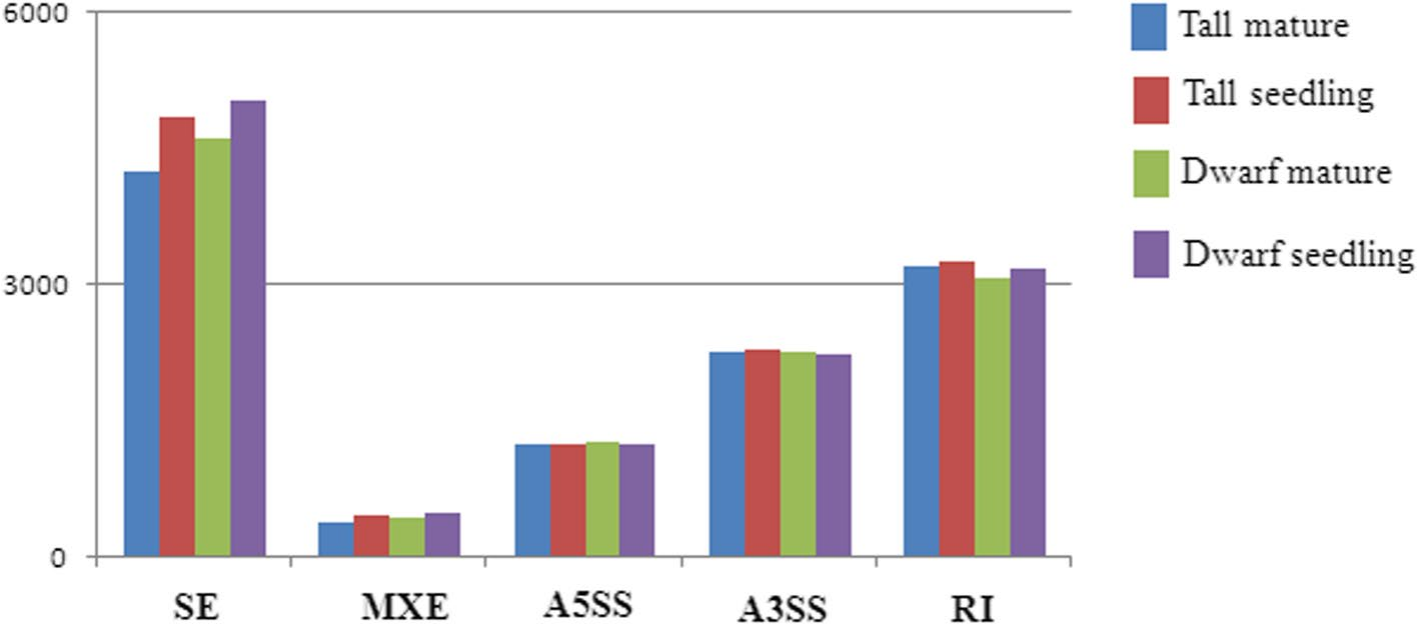
The statistics of alternative splicing types in mature and seedling period from tall and dwarf coconut.

A total of 11,376, 12,124, 11,676, and 12,186 novel transcripts were detected in mature tall coconut trees, tall coconut seedlings, mature trees of dwarf coconut, and dwarf coconut seedlings, respectively. These transcripts were analysed in conjunction with gene predictions to determine if alternative splicing events were occurring. We detected five types of alternative splicing: alternative 3′ splicing sites (A3SS), alternative 5′ splicing sites (A5SS), mutually exclusive exons (MXE), retained introns (RI), and skipped exons (SE). The largest proportion of novel alternatively spliced transcripts were generated by skipped exons (SE), followed by retained introns (RI) and alternative 3′ splicing sites (A3SS), with the fewest number of alternatively spliced transcripts generated by mutually exclusive exons (MXE) (Fig. 6).

**Figure 7:**
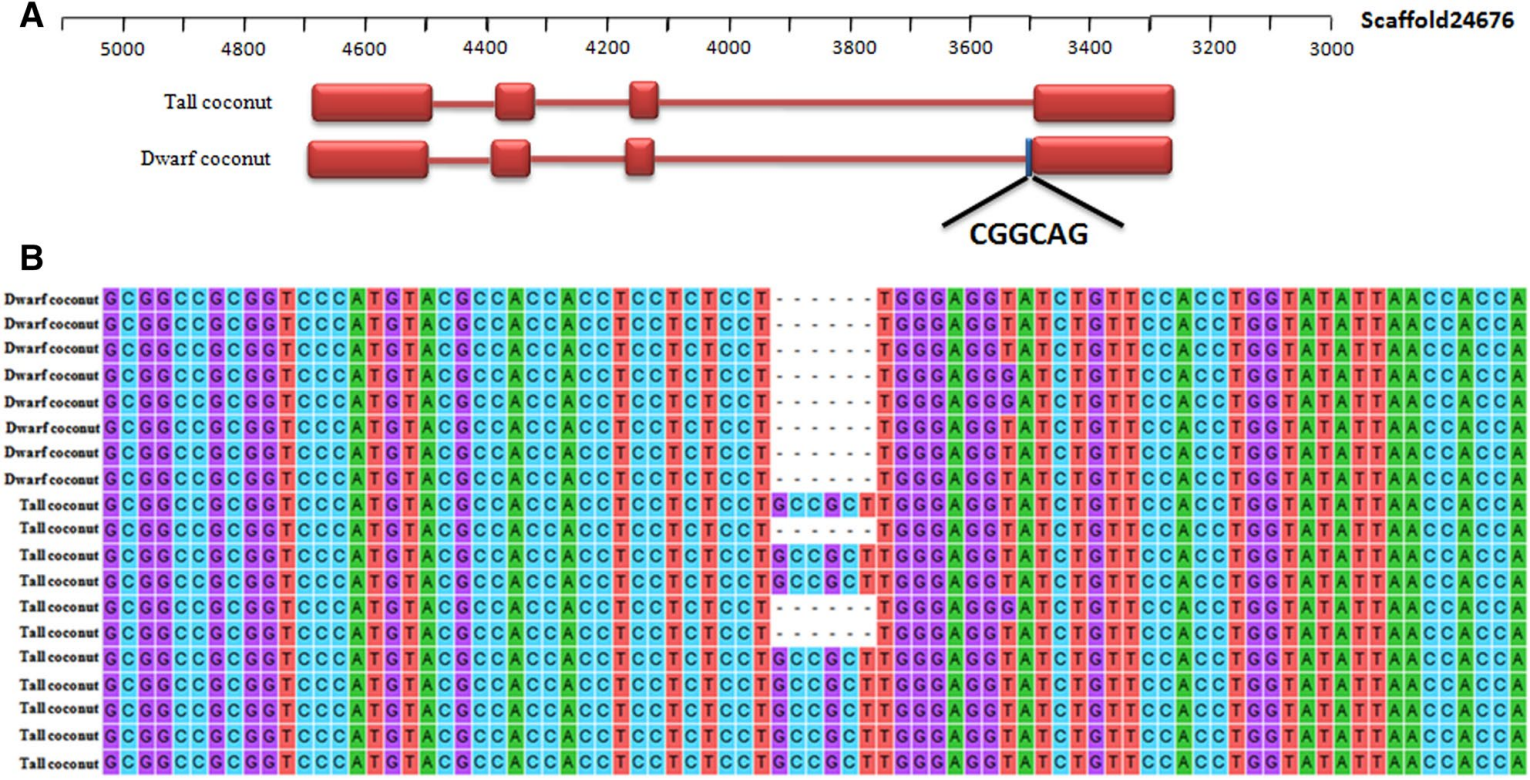
Alternative splicing of the *FT* gene. (**A**) Gene structure of two alternative splice transcripts of *FT* gene. (**B**) Detection of alternative splice of *FT* gene in 8 dwarf coconut and 11 tall coconut samples by cDNA PCR amplification and then sequence.

Meanwhile, we examined in detail any alternative splicing events involving genes in the floweing time pathway. For *FT*, different transcripts were detected between tall and dwarf coconut. Due to alternatice 3′ splicing site (A3SS) in the fourth exon of *FT*, one six-nucleotide indel was detected between tall and dwarf coconut (Fig. 7A). In order to validate the relationship between the six-nucleotide indel and flowering time, we examined *FT* transcripts from 8 tall coconuts and 11 dwarf coconuts. Shorter transcripts (including the six-nucleotide deletion) were detected in all dwarf coconut samples, while 8/11 tall coconut samples showed longer transcripts (without the six-nucleotide deletion) (Fig. 7B and Supplementary File 2).

## Discussion

Coconut (*Cocos nucifera*, 2n = 32), belonging to the genus *Cocos* in the monocotyledonous family Arecaceae (Palmaceae), is an important tropical oil and fruit crop. Coconut can be subdivided into two types according to morphological characteristics: tall coconuts, which flower in 8–10 years, and dwarf coconut, which only need 3–5 years to flower^36^. In order to elucidate the molecular mechanisms underlying differences in flowering time between tall and dwarf coconut, we compared transcriptome data from seedlings and mature trees of tall and dwarf coconuts. We found flowering in *Cocos nucifera* to be putatively regulated by the photoperiod pathway. In this pathway, *FT* is a crucial gene involved in signal transduction; overexpressing *FT* can result in an extremely early-flowering phenotype^7^. In our study, we detected alternative splicing of the *FT* gene between tall and dwarf coconut, as well as a number of key genes putatively involved in regulation of flowering time in coconut.

One of the most crucial phases in flowering plant development is the decision to flower, and flowering time has a major influence on plant fitness. The transition from the vegetative to the reproductive stage is controlled by several external factors, including photoperiod^14^, temperature, and abiotic stress, as well as by internal factors such as hormone production, C/N nutrient ratios and the age of the plant^37^. A complex set of interactions between the internal and external environment together decide when the plant transforms from the vegetative to the reproduction stage. In woody plants, it is difficult to investigate the molecular mechanisms controlling flowering time, due to their long life-cycles. Woody plants, due to their characteristic long life cycles and perenniality, generally have specific strategies to adapt to the ever-changing environment, such as bud dormancy. Despite this, several studies have shown a high level of conservation of genetic networks regulating flowering time between woody and herbaceous plants^38–40^. In our study, some coconut genes also showed high similarity with *Arabidopsis* genes known to be involved in the regulation of flowering time, including *CRY1*, *PHYA*, *PHYB*, *ZTL*, *SOC1*, *ELF3*, *FKF1*, *ELF4*, *CO*, and *FT* homologs. *CRY1* has been validated to up-regulate expression of clock genes in the *Arabidopsis* photoperiod pathway, including *ZTL* (*zeitlupe*), *SOC1* (*suppressor of overexpression of constans*), *ELF3* (*early flowering 3*), *FKF1* (*flavin binding F-box 1*), and *ELF4* (*early flowering 4*)^11^. By contrast, *PHYA* and *PHYB* can suppress the expression of clock genes in the *Arabidopsis* photoperiod pathway^20^. We also observed SOC1 genes were up-regulated expressed in mature period compared to seedling period from tall and dwarf coconut.

Coconut is mainly cultivated in the tropical and subtropical regions, including Central and South America, East and West Africa, Southeast Asia and the Pacific Islands. Hence, it is not possible that flowering time could be regulated by the vernalization pathway in coconut. Based on our transcriptome data, we think that flowering time in coconut is most probably regulated by the photoperiod pathway. We observed high levels of expression of some crucial genes involved in the photoperiod pathway at the mature tree stage compared to the seedling stage.

Most genes in flowering plants contain introns which will be spliced from pre-mRNA in the nucleus before mature mRNA is formed. Splice site variation in the junction between introns and exons will result in multiple mRNA transcripts or isoforms from a single gene^41^. Alternative splicing can contribute to gene regulation and proteome diversity, and is also known to be involved in stress response^42–45^, development and reproductive growth^46–48^. In *Arabidopsis*, *FT* plays an important role in the transition from vegetative growth to reproduction growth^49,50^. Alternative splicing of *FT* in *Chrysanthemum morifolium* results in one *FT* gene encoding up to four different transcripts^51^. The expression level of these four *FT* isoforms depends on the developmental stage of flower, with alternative splicing associated with the degree of early flowering^51^. We also detected alternative splicing of a *CnFT* gene between tall and dwarf coconut. In dwarf coconut, which requires approximately 3–5 years to enter its reproductive stage, we detected only one transcript type, which lacked six nucleotides compared to the transcript type found to be most abundant in tall coconut (which requires 8–10 years to enter its reproductive stage). These results suggest that the *FT* transcript lacking the six nucleotides produce by A3SS may have a positive contribution to the early flowering phenotype in coconut. The other isoform of *CnFT* was detected in 8/11 tall coconut individuals: the remaining three may potentially result from open-pollination/heterozygosity resulting from cross-pollination with dwarf coconut varieties. Our results suggest that this alternative splice variant of *FT* could be a major player in flowering time differentiation between the dwarf and tall coconut types, but further investigation and validation is warranted to confirm this effect.

## Supplementary information

Supplementary Information 1 (PDF 398 kb)

Supplementary Information 2 (PDF 443 kb)

Supplementary Table 1 (XLSX 26 kb)

Supplementary Table 2 (XLSX 22 kb)

## Data Availability

The raw data of transcriptome is from seedling and mature period of tall and dwarf coconut available in the European Nucleotide Archive (Submission Number: PRJEB34852). The CDS and genome sequence (the first version of coconut genome) is available in the GigaDB database (GigaDB, RRID:SCR 004002, https://gigadb.org/dataset/100347)^35^.
