# Supplementary material for: Alternative splicing of flowering time gene FT is associated with halving of time to flowering in coconut
Source: Sci Rep. 2020 Jul 15;10:11640. doi: 10.1038/s41598-020-68431-2 (PMC7363896; doi:10.1038/s41598-020-68431-2)
Supplement: Supplementary file 2 — Supplementary Information 2 (PDF 443 kb) [file 41598_2020_68431_MOESM2_ESM.pdf]

Alternative splicing of flowering time gene *FT* is associated with halving of time to flowering in coconut

Wei Xia[2]*, Rui Liu[1]*, Jun Zhang[1]*, Annaliese S. Mason[3], Zhiying Li[1], Shufang Gong[1], Yazhu Zhong[1], Yajing Dou[1], Xiwei Sun[1], Haikuo Fan[1&], Yong Xiao[1&]

[1] Coconut Research Institute, Chinese Academy of Tropical Agricultural sciences, Wenchang, P.R. China

[2] College of tropical crops, Hainan University, Haikou, P.R. China

[3] Department of Plant Breeding, IFZ Research Centre for Biosystems, Land Use and Nutrition, Justus Liebig University Giessen, Heinrich-Buff-Ring 26-32, 35392i Giessen, Germany

>CCG011565-dwarf-CDS

ATGGCTAGCTGCGTGGATCCCCTTGTGGTAGGGAGGGTCATAGGCGAGGTGGTGGACCTTTTTGTTCCCAC
CATGAGCATGTCTGTGAGCTACGGAACGAAGCATGTCAACAACGGCTGTGACGTGAAGCCGTCCATGGCTA
TCAACCCTCCAACTGTCCAGATCGCAGGCCGACGAGCCGACCTCTATACTCTGGTGATGACCGATCCGGATG
CCCCCAGTCCTAGTGACCCCACTATGAGGGAGTGGCTCCACTGGGTGGTGGTTAATATACCAGGTGGAACA
GATACCTCCCAAG------GAGAGGAGGTGGTGGCGTACATGGGACCGCGGCCGCCGGTGGGGATCCACCGC
TATGTCCTGGTGCTGTTCCAGCAGAAGTCAAGGTTCTTAACGGGGGCGGGGGTGGCTCTGCCTGCGTCTCG
TGCCAACTTCAACACCCGCACCTTCGCCGCCCGGCATGACCTTGGTCTCCCCGTCGCTGCGGTCTACTTCAAT
GCCCAGAAGGAGCCCGCCAACCGCCGCCGGTGA

> CCG011565-tall-CDS

ATGGCTAGCTGCGTGGATCCCCTTGTGGTAGGGAGGGTCATAGGCGAGGTGGTGGACCTTTTTGTTCCCAC
CATGAGCATGTCTGTGAGCTACGGAACGAAGCATGTCAACAACGGCTGTGACGTGAAGCCGTCCATGGCTA
TCAACCCTCCAACTGTCCAGATCGCAGGCCGACGAGCCGACCTCTATACTCTGGTGATGACCGATCCGGATG
CCCCCAGTCCTAGTGACCCCACTATGAGGGAGTGGCTCCACTGGGTGGTGGTTAATATACCAGGTGGAACA
GATACCTCCCAAGCGGCAGGAGAGGAGGTGGTGGCGTACATGGGACCGCGGCCGCCGGTGGGGATCCAC
CGCTATGTCCTGGTGCTGTTCCAGCAGAAGTCAAGGTTCTTAACGGGGGCGGGGGTGGCTCTGCCTGCGTC
TCGTGCCAACTTCAACACCCGCACCTTCGCCGCCCGGCATGACCTTGGTCTCCCCGTCGCTGCGGTCTACTTC
AATGCCCAGAAGGAGCCCGCCAACCGCCGCCGGGAA

Supplementary file 2 Two transcripts sequences of *FT* gene (CCG011565.1)
